# The Association of Serum Levels of Brain-Derived Neurotrophic Factor with the Occurrence of and Recovery from Delirium in Older Medical Inpatients

**DOI:** 10.1155/2017/5271395

**Published:** 2017-02-09

**Authors:** John Williams, Karen Finn, Vincent Melvin, David Meagher, Geraldine McCarthy, Dimitrios Adamis

**Affiliations:** ^1^Pathology Department, Sligo University Hospital Sligo, Sligo, Ireland; ^2^School of Biological Science, Cork Institute of Technology, Cork, Ireland; ^3^Sligo Mental Health Services, Clarion Rd, Sligo, Ireland; ^4^Cognitive Impairment Research Group (CIRG), Graduate Entry Medical School, University of Limerick, Limerick, Ireland; ^5^Sligo Medical Academy, NUI Galway and Sligo Mental Health Services, Clarion Rd, Sligo, Ireland; ^6^Research and Academic Institute of Athens, Athens, Greece

## Abstract

Limited studies of the association between BDNF levels and delirium have given inconclusive results. This prospective, longitudinal study examined the relationship between BDNF levels and the occurrence of and recovery from delirium. Participants were assessed twice weekly using MoCA, DRS-R98, and APACHE II scales. BDNF levels were estimated using an ELISA method. Delirium was defined with DRS-R98 (score > 16) and recovery from delirium as ≥2 consecutive assessments without delirium prior to discharge. We identified no difference in BDNF levels between those with and without delirium. Excluding those who never developed delirium (*n* = 140), we examined the association of BDNF levels and other variables with delirium recovery. Of 58 who experienced delirium, 39 remained delirious while 19 recovered. Using Generalized Estimating Equations models we found that BDNF levels (Wald *χ*^2^ = 7.155; df: 1, *p* = 0.007) and MoCA (Wald *χ*^2^ = 4.933; df: 1, *p* = 0.026) were associated with recovery. No significant association was found for APACHE II, dementia, age, or gender. BDNF levels do not appear to be directly linked to the occurrence of delirium but recovery was less likely in those with continuously lower levels. No previous study has investigated the role of BDNF in delirium recovery and these findings warrant replication in other populations.

## 1. Introduction

Delirium is a neuropsychiatric syndrome characterized by abrupt disturbance in attention, awareness, consciousness, and cognition. It is frequently accompanied by a variety of behavioural disturbances, altered psychomotor activity, disruption of sleep-wake cycle, and psychosis. These disturbances develop over a short period and tend to fluctuate. [1]. Although delirium has classically been characterized as a highly reversible and relatively transient phenomenon, accumulating evidence indicates that in many cases it persists and is associated with serious adverse longer-term outcomes (including cognitive and functional deterioration, high rates of mortality, and prolonged hospitalisation) especially in older populations [2, 3]. Delirium often occurs in the context of infectious illness and thus a number of studies have investigated cytokine levels in an effort to illuminate the pathophysiology of delirium, but with inconclusive and conflicting results [4]. In contrast, previous work [5] found that low levels of neuroprotective factors (IGF-I and IL-1RA) were associated with delirium.

Brain-Derived Neurotrophic Factor (BDNF) is a mediator of neuronal development [6] that promotes the survival of neurons [7] and is important for cell proliferation [8] and inflammatory processes [9, 10]. It has been reported that BDNF is associated with the pathogenesis of several neuropsychiatric disorders such as schizophrenia [11], depression [12], Alzheimer's disease [13], Huntington's chorea [14], and alcohol withdrawal delirium [15].

Few studies have investigated BDNF levels in relation to delirium with conflicting and inconclusive results. A study in ICU patients [16] found that serum BDNF levels were significantly higher in delirious patients compared to nondelirious controls. In contrast, a study in oncology inpatients [17] did not find any significant differences in the serum levels of BDNF between delirious and nondelirious patients. Similarly, Tomasi et al. [18] in a study of community-acquired pneumonia found that patients “with sepsis-associated encephalopathy (SAE)” had higher levels of BDNF compared to “delirious patients.” Another study examining outcomes in ICU patients reported that low levels of BDNF were associated with increased mortality [19]. The pathophysiology of delirium has been understudied [20] and, in particular, it remains uncertain how such a wide range of potential aetiologies, many of which involve pathology at peripheral body locations and without clear links to CNS function, can produce such a consistent complex neuropsychiatric picture that reflects generalized brain dysfunction. Studies of prevalent delirium may miss associations that are important during the early/developing phases of medical illness and we therefore decided to explore the BDNF-delirium relationship across the entire hospital stay of a group of older medically ill people, in which there would be both prevalent and incident delirium and to follow this to define course as either recovering or continuous. Therefore, the aims of the present study were (a) to investigate the relation of BDNF to the occurrence of delirium (both incidence and prevalence) and (b) the association of BDNF levels along with other relevant factors in the recovery of delirium.

## 2. Materials and Methods

### 2.1. Design of the Study

The study is a prospective longitudinal observational study.

### 2.2. Setting Inclusion/Exclusion Criteria

All acute medical admissions of patients 70 years old and over to a University Hospital in Sligo in the northwest of Ireland were approached to enter the study within 72 hours of admission. Patients who were admitted twice during the study period were included only on their first admission. Patients who were in a terminal phase of illness, with severe aphasia, intubated, with severe sensory problems, or unable to speak English were excluded from the study.

### 2.3. Clinical Assessments: Measurements Scales

Demographics (gender, age, years of education, marital status, and living circumstances) were collected from the medical case notes and from the hospital computerised database.

At each assessment, the following were carried out:

#### 2.3.1. Cognitive Assessment

Montreal Cognitive Assessment (MoCA) [21] was used to assess general cognition. Administration typically takes about 12–15 min. The MoCA is scored on a 30-point scale. Higher scores indicate better cognitive performance. If patients could not complete sections (e.g., due to visual impairment) it was standardized to give a maximum score of 30.

#### 2.3.2. Delirium Assessment

Delirium severity and presence/absence were measured using the Revised Delirium Rating Scale (DRS-R-98) [22], a 16-item rating scale with thirteen severity items and 3 diagnostic items. The 13-item severity section can be scored separately from the 3-item diagnostic section. The DRS-R98 severity scale score ranges from 0–39 with higher scores indicating more severe delirium [22]. A score of 16 or more is compatible with the diagnosis of delirium. We defined as having* delirium* those with a score of 16 and above in DRS-R98 severity score (categorical variable yes/no) and consequently as* recovered* from delirium those who did not have delirium for at least two assessments before discharge [23].

#### 2.3.3. Severity of Physical Illness

The Acute Physiology and Chronic Health Evaluation II (APACHE II) [24] and its subscale Acute Physiology Score (APS) were used to measure severity of physical illness. This includes age, chronic illness, and acute physiological disturbance. The latter is measured with its subscale APS which has 11 items and each one can be scored from 0 to 4 (4 is the worst). The range of APACHE II is from 0 to 71 and an increased score is closely correlated with more severe illness and subsequent risk of hospital death.

#### 2.3.4. Diagnosis of Previous History of Dementia

Cases with preexisting dementia were identified as a clear history of documented DSM-IV diagnosis dementia or by using the Short Informant Questionnaire on Cognitive Decline (IQCODE) with a cut-off point of ≥3.5. [25]

### 2.4. Procedures

All eligible and consenting patients had an assessment at first recruitment day. Thereafter 7 more assessments were carried out every 3 (±1) days if patients were in hospital. The maximum number of assessment was thus eight. Venous samples of blood (nonfasting) were taken on the same day of each assessment, centrifuged immediately (maximum elapsed time 20 minutes), to avoid release of BDNF from platelets, and stored at −70°C until analysis. Levels of BDNF were estimated with the ELISA method Promega BDNF Emax(R) immunoassay system and are expressed in ng/mL.

### 2.5. Ethics

Informed consent was obtained using a previously published method [26]. Consent was obtained separately for the withdrawn blood. The study was approved by the Sligo University Hospital Research Ethics Committee.

### 2.6. Statistical Analysis

Data were analysed with SPSS v20. Descriptive statistics are presented as means or counts and percentages. To examine any significant effects of BDNF, cognition, and the other measurements on delirium status, the Generalized Estimating Equations (GEE) method was used. This model takes into account all the data, allows for missing values, and adjusts for correlations due to repeated assessments of each participant [27]. Because the dependent variables were categorical the binominal distribution with logit link was used. When the dependent variable was time-variant (e.g., delirium/no delirium) the “unstructured” working correlation matrix was assumed and when the dependent variable was time-invariant the “independent” working correlation matrix was assumed. The best fitting model was taken as the one with the lowest value of Corrected Quasi-Likelihood under Independence Model Criterion (QICC).

## 3. Results

### 3.1. Descriptive Statistics of the Sample

The sample consisted of 198 participants (mean age 80.63; SD: 6.81; range 70–97). Of these 92 (46.5%) were females. Eighty-six (43.4%) were identified with a history of dementia.  Table 1 shows the clinical characteristics of those with and without delirium at each assessment, including ratings for the MoCA, APACHE II, and BDNF levels at each assessment.

### 3.2. Missing Values

Evaluation of missing values using Little's MCAR test indicated that they were missing completely at random (MCAR *χ*^2^ = 12.24, df = 9, *p* = 0.20).

### 3.3. Examination of the Effects of Potential Explanatory Variables on the Presence or Absence of Delirium (Longitudinal Analysis)

Treating the subjects as random effects and the number of assessments as repeated measurements, we generated an initial GEE model (binomial, link function logit) with the dependent variable as the presence or not of delirium (as defined with the DRS-R98) at any assessment and as independent variables the levels of BDNF, APACHE II, MoCA scores, age, gender, and previous history of dementia. After fitting different models by systematically removing variables with nonsignificant effects and comparing the QICCs a parsimonious model was achieved with the lowest QICC value (Table 2).

Significant predictors for the presence of delirium were previous history of dementia, older age, lower scores on MoCA, and more severe physical illnesses as measured with the APACHE II. Levels of BDNF did not have any significant effect on the occurrence of delirium (Table 2).

### 3.4. Examination of the Effects of Explanatory Variables on the Recovery of Delirium (Longitudinal Analysis)

**Figure 1:**
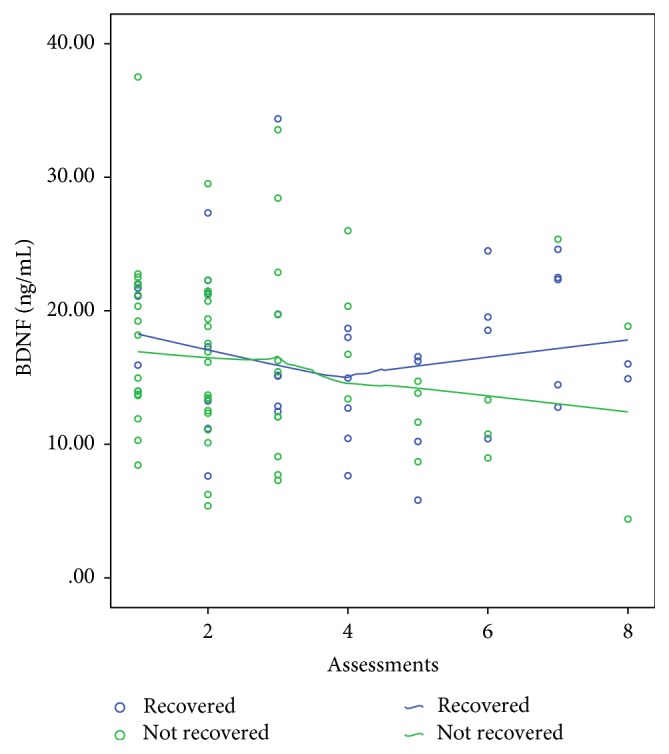
BDNF levels: scatterplot of levels of BDNF across the time between those who recovered from delirium and those who did not. Fitted lines with Loess Kernel Epanechnikov method (90% points fitted).

**Figure 2:**
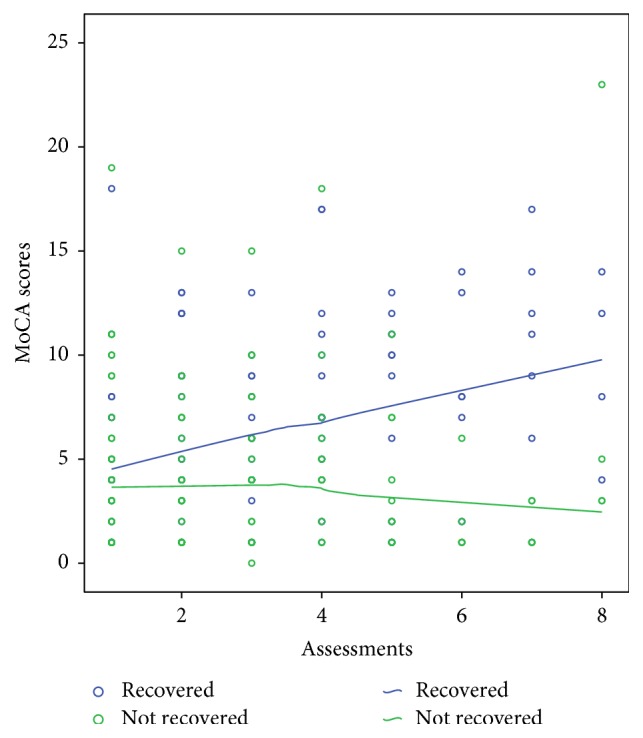
MoCA scores across the time for those who recovered from delirium and those who did not. Fitted lines with Loess Kernel Epanechnikov method (90% points fitted).

During the observation period 140 participants (70.7 %) never developed delirium. From the remaining 58, 19 (32.8%) recovered from delirium (i.e., evidenced by the final two or more assessments being negative for delirium). After excluding those who never developed delirium, we conducted a similar analysis with the GEE model as above but with recovery or not from delirium as the dependent variable and with the levels of BDNF, APACHE II, and MoCA scores, age, gender, and previous history of dementia as independent variables. The final most parsimonious model is presented in Table 3 where it can be seen that, after controlling for the other variables, low levels of BDNF and higher MoCA scores were the only independent predictors for delirium recovery. Figures 1 and 2 depict a graphic illustration of the levels of BDNF and MoCA scores across the time between those who recovered and those who did not recover from delirium using fitted lines as per the Loess Kernel Epanechnikov method (90% points fitted). Regarding the BDNF (Figure 1), those who did not recover had persistently lower levels of BDNF compared to those who recovered. Regarding the MoCA scores (Figure 2) those who recovered had steadily increasing scores during the assessments.

## 4. Discussion

This longitudinal study confirms that delirium status is associated with cognitive impairment as measured by the MoCA, previous history of dementia, advanced age, and severity of physical illness, in keeping with previous work [28]. We did not find any significant association between BDNF levels and delirium (incident or prevalent). In the few previous studies that have been conducted there have been variable findings; Brum et al. [17] did not find any significant differences in the serum levels of BDNF between delirious and nondelirious oncology patients, while Grandi et al. [16] reported that serum BDNF levels were significantly higher in delirious patients than in nondelirious controls in an ICU setting. Given that BDNF is a neurotrophic factor it might be expected that the occurrence of delirium would if anything be linked to lower levels [29]. In keeping with this assertion, lower levels of BDNF are associated with delirium tremens [15]. However a direct comparison cannot apply as these two conditions have phenomenological and aetiological differences; BDNF levels are increased in vitro and in vivo following acute alcohol exposure but are reduced with chronic use [30] and genetic variants are associated with alcohol withdrawal delirium [31].

In addition, the results from the present study indicate that lower initial levels of BDNF together with higher MoCA scores are associated with a reduced frequency of delirium recovery. Figure 2 depicts how an initial drop in BDNF levels is followed by an increase in those who recover but not in those who do not. No previous studies have investigated the role of BDNF in recovery of delirium. It was reported that the levels of BDNF were consistently low only in a case report of a patient with systemic lupus erythematosus who had continuous delirium [32]. Although normative data do not exist (as different kits and different methods of evaluation of the levels of BDNF produce different “normal values”) previous research suggests that levels of BDNF are slow to normalise [33]. Therefore we cannot conclude from our findings that BDNF levels are normalised after delirium recovery. However, a similar pattern exists for other neuroprotective factors such as IGF-I [34] in which delirious patients with lower initial levels of IGF-I were also those who finally recovered from delirium. It appears that a feedback mechanism maintains levels of neuroprotective factors above a minimum, and in those with delirium who eventually recover the levels of neuroprotective factors steadily increase over time [35].

We investigated levels of BDNF in peripheral blood. This is likely to reflect levels of BDNF in the brain since studies have shown that BDNF crosses the blood-brain barrier and that BDNF levels in the brain and serum are closely correlated [36, 37].

The present study, to our knowledge, is the first study to investigate BDNF levels in delirium in older medical inpatients and explore the association with recovery of delirium. This lack of data makes comparison difficult and limits efforts to formulate hypotheses around pathophysiological mechanisms. Therefore, and in view of our relatively small sample size, these results need to be interpreted with caution.

A limitation of this study is that patients were followed only until their discharge from hospital, so the categorisation of patients as recovered or not recovered from delirium is compounded by other factors that resulted in patients' overall clinical recovery or social circumstances allowing their discharge from hospital. Finally, the study involved only hospitalised patients, thus excluding patients with delirium in the context of illness not resulting in hospital admission.

## 5. Conclusions

We did not find any association of BDNF levels with the occurrence of delirium but identified an association with recovery from delirium. If these findings are confirmed in further studies then this would indicate that BDNF could be used as a marker of recovery. Therefore, this suggests that recovery from delirium may be predicted based on biological variables, which in turn has important clinical implications for the prognosis and treatment of delirium.

## Figures and Tables

**Table 1:** Cases of delirium (according to DRS-R98 > 16) with ratings of the MoCA, APACHE II, and BDNF levels (ng/mL) at each assessment point.

Assessments		MOCA	APACHE II	BDNF (ng/mL)
(1)				
No delirium	Mean	12.11	8.25	18.01
	SD	7.66	3.30	7.35
	Valid *N*	157	162	67
Delirium	Mean	2.85	10.46	19.63
	SD	2.61	4.79	3.62
	Valid *N*	34	35	11
(2)				
No delirium	Mean	11.52	8.48	16.43
	SD	8.22	3.58	7.79
	Valid *N*	117	123	41
Delirium	Mean	3.12	10.11	17.94
	SD	2.47	4.13	4.75
	Valid *N*	26	27	13
(3)				
No delirium	Mean	9.94	8.77	16.80
	SD	7.88	3.70	7.41
	Valid *N*	72	74	25
Delirium	Mean	4.48	9.32	13.47
	SD	3.08	3.22	3.64
	Valid *N*	21	25	10
(4)				
No delirium	Mean	11.30	8.55	15.96
	SD	7.48	3.44	7.67
	Valid *N*	50	51	17
Delirium	Mean	5.54	9.73	17.84
	SD	3.13	3.37	5.48
	Valid *N*	13	15	5
(5)				
No delirium	Mean	9.38	8.41	18.28
	SD	7.16	3.05	6.74
	Valid *N*	39	41	10
Delirium	Mean	2.20	10.64	11.69
	SD	1.93	3.56	3.01
	Valid *N*	10	11	3
(6)				
No delirium	Mean	5.87	9.11	15.46
	SD	4.94	3.38	6.55
	Valid *N*	23	27	7
Delirium	Mean	2.50	13.20	12.05
	SD	2.38	4.76	1.81
	Valid *N*	4	5	2
(7)				
No delirium	Mean	8.33	8.82	16.38
	SD	6.99	3.80	5.88
	Valid *N*	21	22	10
Delirium	Mean	1.00	11.00	.
	SD	.	.	.
	Valid *N*	1	1	0
(8)				
No delirium	Mean	11.78	7.78	15.15
	SD	7.07	4.12	.78
	Valid *N*	9	9	3
Delirium	Mean	3.67	8.67	11.62
	SD	1.15	2.89	10.21
	Valid *N*	3	3	2

**Table 2:** GEE model examining the effects of independent predictor variables on delirium status.

Parameter	*B*	Std. error	95% Wald confidence interval		Hypothesis test		
			Lower	Upper	Wald chi-square	df	Sig.
Intercept	7.82	2.5763	2.773	12.872	9.219	1	.002
Prev hx of dementia = No	.89	.3214	.266	1.526	7.770	1	.005
Prev hx of dementia = yes	0	.	.	.	.	.	.
Age	−.086	.0285	−.142	−.030	9.075	1	.003
MoCA	.210	.0257	.160	.260	66.959	1	.000
APACHE II	−.096	.0380	−.170	−.021	6.360	1	.012

**Table 3:** Parameter estimates and significant effects of independent variables on delirium recovery.

Parameter	*B*	Std. error	95% Wald confidence interval		Hypothesis test		
			Lower	Upper	Wald chi-square	df	Sig.
MoCA	.069	.0311	.008	.130	4.933	1	.026
BDNF	−.045	.0169	−.078	−.012	7.155	1	.007

## References

[1] APA (2013). *Diagnostic and Statistical Manual of Mental Disorders DSM- 5*.

[2] Adamis D., Meagher D., Treloar A. (2014). Phenomenological and biological correlates of improved cognitive function in hospitalized elderly medical inpatients. *Archives of Gerontology and Geriatrics*.

[3] MacLullich A. M. J., Beaglehole A., Hall R. J., Meagher D. J. (2009). Delirium and long-term cognitive impairment. *International Review of Psychiatry*.

[4] Adamis D., Preedy V. R., Hunter R. (2011). Cytokines in the elderly. *Cytokines*.

[5] Adamis D., Lunn M., Martin F. C. (2009). Cytokines and IGF-I in delirious and non-delirious acutely ill older medical inpatients. *Age and Ageing*.

[6] Barde Y., Aebischer P., Hefti F. (1999). Biological roles of neurotrophins. *Neurotrophic Factors*.

[7] Linnarsson S., Willson C. A., Ernfors P. (2000). Cell death in regenerating populations of neurons in BDNF mutant mice. *Molecular Brain Research*.

[8] Tessarollo L. (1998). Pleiotropic functions of neurotrophins in development. *Cytokine & Growth Factor Reviews*.

[9] Gillardon F., Eschenfelde C., Rush R. A., Zimmerman M. (1995). Increase in neuronal jun immunoreactivity and epidermal ngf levels following uv exposure of rat skin. *NeuroReport*.

[10] Vega J. A., García-Suárez O., Hannestad J., Pérez-Pérez M., Germanà A. (2003). Neurotrophins and the immune system. *Journal of Anatomy*.

[11] Hori H., Yoshimura R., Yamada Y. (2007). Effects of olanzapine on plasma levels of catecholamine metabolites, cytokines, and brain-derived neurotrophic factor in schizophrenic patients. *International Clinical Psychopharmacology*.

[12] Yoshimura R., Mitoma M., Sugita A. (2007). Effects of paroxetine or milnacipran on serum brain-derived neurotrophic factor in depressed patients. *Progress in Neuro-Psychopharmacology and Biological Psychiatry*.

[13] Laske C., Stransky E., Leyhe T. (2006). Decreased brain-derived neurotrophic factor (BDNF)- and ß-thromboglobulin (*β*-TG)- blood levels in Alzheimer's disease. *Thrombosis and Haemostasis*.

[14] Ciammola A., Sassone J., Cannella M. (2007). Low brain-derived neurotrophic factor (BDNF) levels in serum of Huntington's disease patients. *American Journal of Medical Genetics Part B: Neuropsychiatric Genetics*.

[15] Huang M.-C., Chen C.-H., Liu H.-C., Chen C.-C., Ho C.-C., Leu S.-J. (2011). Differential patterns of serum brain-derived neurotrophic factor levels in alcoholic patients with and without delirium tremens during acute withdrawal. *Alcoholism: Clinical and Experimental Research*.

[16] Grandi C., Tomasi C. D., Fernandes K. (2011). Brain-derived neurotrophic factor and neuron-specific enolase, but not S100*β*, levels are associated to the occurrence of delirium in intensive care unit patients. *Journal of Critical Care*.

[17] Brum C., Stertz L., Borba E., Rumi D., Kapczinski F., Camozzato A. (2015). Association of serum brain-derived neurotrophic factor (BDNF) and tumor necrosis factor-alpha (TNF-*α*) with diagnosis of delirium in oncology inpatients. *Revista Brasileira de Psiquiatria*.

[18] Tomasi C. D., Vuolo F., Generoso J. (2016). Biomarkers of delirium in a low-risk community-acquired pneumonia-induced sepsis. *Molecular Neurobiology*.

[19] Ritter C., Miranda A. S., Giombelli V. R. (2012). Brain-derived neurotrophic factor plasma levels are associated with mortality in critically ill patients even in the absence of brain injury. *Critical Care*.

[20] Meagher D. (2009). More attention, less confusion: time to lessen the burden of delirium. *International Review of Psychiatry*.

[21] Nasreddine Z. S., Phillips N. A., Bédirian V. (2005). The montreal cognitive assessment, MoCA: a brief screening tool for mild cognitive impairment. *Journal of the American Geriatrics Society*.

[22] Trzepacz P. T., Mittal D., Torres R., Kanary K., Norton J., Jimerson N. (2001). Validation of the delirium rating scale-revised-98: comparison with the delirium rating scale and the cognitive test for delirium. *Journal of Neuropsychiatry and Clinical Neurosciences*.

[23] Adamis D., Devaney A., Shanahan E., McCarthy G., Meagher D. (2015). Defining ‘recovery’ for delirium research: a systematic review. *Age and Ageing*.

[24] Knaus W. A., Draper E. A., Wagner D. P., Zimmerman J. E. (1985). APACHE II: a severity of disease classification system. *Critical Care Medicine*.

[25] Jorm A. F. (1994). A short form of the informant questionnaire on cognitive decline in the elderly (IQCODE): development and cross-validation. *Psychological Medicine*.

[26] Adamis D., Martin F. C., Treloar A., Macdonald A. J. D. (2005). Capacity, consent, and selection bias in a study of delirium. *Journal of Medical Ethics*.

[27] Adamis D. (2009). Statistical methods for analysing longitudinal data in delirium studies. *International Review of Psychiatry*.

[28] Ahmed S., Leurent B., Sampson E. L. (2014). Risk factors for incident delirium among older people in acute hospital medical units: a systematic review and meta-analysis. *Age and Ageing*.

[29] Cerejeira J., Firmino H., Vaz-Serra A., Mukaetova-Ladinska E. B. (2010). The neuroinflammatory hypothesis of delirium. *Acta Neuropathologica*.

[30] Cavus S. Y., Dilbaz N., Darcin A. E., Eren F., Kaya H., Kaya O. (2012). Alterations in serum BDNF levels in early alcohol withdrawal and comparison with healthy controls. *Bulletin of Clinical Psychopharmacology*.

[31] Adamis D., Van Munster B. C., Macdonald A. J. D. (2009). The genetics of deliria. *International Review of Psychiatry*.

[32] Ikenouchi-Sugita A., Yoshimura R., Ueda N., Kodama Y., Umene-Nakano W., Nakamura J. (2008). Continuous decrease in serum brain-derived neurotrophic factor (BDNF) levels in a neuropsychiatric syndrome of systemic lupus erythematosus patient with organic brain changes. *Neuropsychiatric Disease and Treatment*.

[33] Chiaretti A., Piastra M., Polidori G. (2003). Correlation between neurotrophic factor expression and outcome of children with severe traumatic brain injury. *Intensive Care Medicine*.

[34] Adamis D., Treloar A., Martin F. C., Gregson N., Hamilton G., Macdonald A. J. D. (2007). APOE and cytokines as biological markers for recovery of prevalent delirium in elderly medical inpatients. *International Journal of Geriatric Psychiatry*.

[35] Adamis D., Meagher D. (2011). Insulin-like growth factor I and the pathogenesis of delirium: a review of current evidence. *Journal of Aging Research*.

[36] Pan W., Banks W. A., Fasold M. B., Bluth J., Kastin A. J. (1998). Transport of brain-derived neurotrophic factor across the blood-brain barrier. *Neuropharmacology*.

[37] Karege F., Schwald M., Cisse M. (2002). Postnatal developmental profile of brain-derived neurotrophic factor in rat brain and platelets. *Neuroscience Letters*.

